# CTCF regulates NELF, DSIF and P-TEFb recruitment during transcription

**DOI:** 10.1080/21541264.2015.1095269

**Published:** 2015-09-23

**Authors:** Clélia Laitem, Justyna Zaborowska, Michael Tellier, Yuki Yamaguchi, Qingfu Cao, Sylvain Egloff, Hiroshi Handa, Shona Murphy

**Affiliations:** 1 Sir William Dunn School of Pathology; University of Oxford; Oxford, UK; 2 Graduate School of Bioscience and Biotechnology; Tokyo Institute of Technology; Yokohama, Japan; 3 Université de Toulouse; UPS; Laboratoire de Biologie Moléculaire Eucaryote; Toulouse, France; 4 Department of Nanoparticle Translational Research; Tokyo Medical University; Tokyo, Japan; # Current address: Immunocore Limited; Milton Park, Abingdon, Oxon, UK

**Keywords:** CTCF, elongation checkpoint, RNA Polymerase II pausing, P-TEFb, snRNA gene, transcription

## Abstract

CTCF is a versatile transcription factor with well-established roles in chromatin organization and insulator function. Recent findings also implicate CTCF in the control of elongation by RNA polymerase (RNAP) II. Here we show that CTCF knockdown abrogates RNAP II pausing at the early elongation checkpoint of *c-myc* by affecting recruitment of DRB-sensitivity-inducing factor (DSIF). CTCF knockdown also causes a termination defect on the U2 snRNA genes (*U2*), by affecting recruitment of negative elongation factor (NELF). In addition, CTCF is required for recruitment of positive elongation factor b (P-TEFb), which phosphorylates NELF, DSIF, and Ser2 of the RNAP II CTD to activate elongation of transcription of *c-myc* and recognition of the snRNA gene-specific 3’ box RNA processing signal. These findings implicate CTCF in a complex network of protein:protein/protein:DNA interactions and assign a key role to CTCF in controlling RNAP II transcription through the elongation checkpoint of the protein-coding *c-myc* and the termination site of the non-coding *U2*, by regulating the recruitment and/or activity of key players in these processes.

## Abbreviations

BRD4: Bromodomain Containing 4
ChIP: Chromatin immunoprecipitation
CTCF: CCCTC binding factor
CDK9: cyclin-dependent protein kinase 9
CKII: casein kinase II
CTD: RNAP II carboxyl-terminal domain
DRB: 5,6-dichlorobenzimidazone-1-β-D-ribofuranoside
DSIF: DRB-sensitivity-inducing factor
GRO-seq: global run on sequencing
GST: glutathione S-transferase
K_D_: knockdown
KSHV: Kaposi's sarcoma-associated herpes virus
IPTG: Isopropyl β-D-1-thiogalactopyranoside
LANA: Latency-Associated Nuclear Antigen
NELF: negative elongation factor
NFkB: Nuclear Factor Kappa B
P-TEFb: positive elongation factor b
PBS: Phosphate-buffered saline
qPCR: quantitative real-time PCR
qRT-PCR: quantitative reverse-transcriptase PCR
RNAP II: RNA polymerase II
snRNA: small nuclear RNA
STX4: Syntaxin 4
TSS: transcription start site
siRNA: small interfering RNA
U2RA: U2read-around
U2RT: U2 read-through
WBP5: WW Domain Binding Protein 5.

## Introduction

CCCTC binding factor (CTCF) is a highly conserved and ubiquitously-expressed transcription factor, which regulates gene expression and organizes chromatin structure.^1^ It is a critical factor for various cellular processes, including growth, proliferation, differentiation, and apoptosis in mammalian cells,^2,3^ and homozygous CTCF knockout mice exhibit early embryonic lethality prior to implantation.^4^

CTCF binding sites are important elements of insulators, which block communication between adjacent regulatory elements and prevent spreading of heterochromatin.^1,5,6^ CTCF also acts at the level of transcription mainly as a repressor, for example at the *c-myc, pax-6*, and chicken lysozyme genes.^7-9^

CTCF is a 727 amino-acid protein with a central zinc-finger DNA binding domain.^7^ Its properties are modulated by post-translational modifications^10^ and interaction with a range of partners.^11^ For instance, the C-terminal domain of CTCF is phosphorylated *in vivo* and this phosphorylation can be recapitulated *in vitro* by casein kinase II (CKII).^10^ The CTCF C-terminal domain is involved in interaction between CTCF and RNA polymerase II (RNAP II) and its phosphorylation decreases the efficiency of this interaction.^12^ El-Kady and Klenova^13^ suggest that phosphorylation converts CTCF from a repressor to an activator.

Approximately 20% of CTCF sites are located within 2 kb of transcription start sites (TSS), suggesting a role for CTCF in the regulation of transcription at the 5′ end of genes.^5,14^ Comparing the data obtained from various studies,^14-19^ Parades et al.^20^ found that CTCF binding at promoter proximal regions is correlated with RNAP II pausing (GRO-seq data).^17^ In agreement with this, CTCF is able to slow RNAP II down in an *in vitro* transcription system.^21^ Accordingly, Shukla et al.^21^ proposed that CTCF binding in the vicinity of intron/exon junctions slows RNAP II down to allow the recruitment of splicing factors.^21^ RNAP II also tends to stall at cohesin/CTCF binding sites in long genes^22^ and in the Latency-Associated Nuclear Antigen (LANA) gene of Kaposi's sarcoma-associated herpes virus (KSHV).^23^ Finally, CTCF is found at RNAP II stalling or termination sites on both protein-coding and snRNA genes.^24^ Taken together, these data indicate that CTCF can play an important role in the regulation of RNAP II stalling/termination. However, the molecular mechanism is not yet clear. RNAP II often stalls soon after transcription initiation at an early elongation checkpoint before the transition to productive elongation.^25,26^ The negative elongation factors, NELF, comprising Nelf-A, Nelf-B, Nelf-C/D, and Nelf-E subunits,^27,28^ and DRB sensitivity-inducing factor DSIF,^29,30^ a heterodimer of Spt4 and Spt5,^28,31,32^ are required to stall RNAP II at the elongation checkpoint on protein-coding genes.^33^ Release from this checkpoint is mediated by positive transcription elongation factor-b (P-TEFb), which comprises CDK9 kinase and cyclin T1. CDK9 phosphorylates the Nelf-E subunit of NELF, the Spt5 subunit of DSIF and Ser2 of the Tyr1/Ser2/Pro3/Thr4/Ser5/Pro6/Ser7 heptapeptide repeat of the C-terminal domain (CTD) of RNAP II.^34-37^ Interestingly, NELF is also involved in termination of transcription of the RNAP II-transcribed non-coding *U2.*^24^ In this case, P-TEFb is not required for transcription but for co-transcriptional recognition of the snRNA gene-specific 3’ end RNA processing element, the 3’ box.^38,39^

To investigate the role of CTCF in RNAP II stalling/termination of transcription, we have analyzed the effect of CTCF knockdown on RNAP II stalling at the early elongation checkpoint of *c-myc*, which occurs within 100 bp of the TSS, and on termination of transcription of *U2*. Our results indicate that CTCF knockdown causes an increase in RNAP II transcription through both the early elongation checkpoint of *c-myc* and the normal transcription termination site of *U2*. CTCF is required for the efficient recruitment or retention of NELF and DSIF at sites of RNAP II stalling/termination. The association of these factors correlates with repression of *c-myc* and efficient termination of transcription of *U2*. CTCF also enhances P-TEFb recruitment, which is required for RNAP II release from the elongation checkpoint on protein-coding genes and for efficient 3’ box-dependent processing of snRNA gene transcripts. In contrast, NELF and DSIF are not required for CTCF binding, indicating that CTCF recruitment initiates a cascade of interactions that lead either to RNAP II stalling followed by the transition to productive elongation (*c-myc*) or to termination of transcription (*U2*). Our data therefore highlights a new function of CTCF as a regulator of RNAP II stalling at the elongation checkpoint of *c-myc* and at the termination site of *U2*. In addition, the interactions between CTCF and NELF, DSIF and P-TEFb we describe provide a molecular mechanism for the effect of CTCF on transcription elongation.

## Materials and Methods

### Cell lines and siRNA-mediated knockdown

HeLa cells were grown in DMEM medium supplemented with 10% fetal calf serum, 100 U/mL penicillin, 100 ug/ml streptomycin and 2 mM L-glutamine at 37ºC and 5% CO2. siRNAs targeting CTCF, the Nelf-E subunit of NELF and the Spt5 subunit of DSIF (Dharmacon siGENOME SMART pool; M-020165-02; M-011761-01; M-016234-01) were transfected using Lipofectamine 2000 (Life Technologies, 11668027) according to the manufacturer's instructions.

### Nuclear extract preparation

HeLa nuclear extracts were produced as described.^40^

### Western blot analysis

Western blot analysis was performed as previously described^38^ using approximately 30 µg of proteins harvested from cells boiled in Laemmli buffer (50mM Tris(pH6.8), 2% sodium dodecyl sulfate, 5% β-mercaptoethanol, 10% glycerol, 0.1% bromophenol blue) and antibodies against CTCF (Millipore, 07 729), Nelf-A (Santa cruz, sc-32911), Nelf-E (Santa cruz, sc32912), RNAP II (Santa cruz, sc-9001), Spt5 (Santa cruz, sc-28678), CDK9 (Santa cruz, sc-484), cyclin T1 (Santa cruz, sc-10750), Rad21 (Abcam, ab992), c-Myc (Abcam, 9106), and α-tubulin (Tebu-Bio, 200-301-880).

### Chromatin Immunoprecipitation

HeLa cells were transfected using Lipofectamine 2000 (Life Technologies, 11668027) before being subjected to ChIP analysis following the protocol from the Farnham Lab^41^ using antibodies against control IgG (Sant cruz, sc-2027), CTCF (Millipore, 07 729), histone H3 (Abcam, ab1791), RNAP II (Santa Cruz, sc-899), NELF-A (Santa Cruz, sc-32911), Spt5 (Santa Cruz, sc-28678), CDK9 (Santa Cruz, sc-8338), cyclin T1 (Santa Cruz, sc-10750), pSer5 (Abcam, 5131) and pSer2 (Abcam, 5095). ChIP samples were analyzed by qPCR using QuantiTect SYBR Green PCR (Qiagen, 204145). Final ChIP values are expressed as a percentage of the total DNA input after deduction of the signal obtained using rabbit IgG as a negative control. Sequences of primer pairs are provided in **Table 1**.

**Table 1.** Sequences of primers used for qPCR and qRT-PCR

Name	Forward	Reverse
U2 snRNA gene		
1	GGAGCGGAGCGTTCTCTGTCT CCCC	AGAGTGTGAGCCCTCATTCACGCCC
2	ATGAGAGTGGGACGGTGA	CACTTGATCTTAGCCAAAAGG
3	ACGAGTCCTGTGACGCGCCGGCTTG	CTCCGGGTGGGTCCCATTCCTTTAA
4	CCTCCCCGCCTCTCCCTCGCTC	GGACAAATAGCCAACGCATGCGG
5	AGATCGCGCCATTGCACTGC	CCCGAACAGGTTTTCACTAGG
c-myc gene		
1	CTCCTGCTCCTGCCCCCACCTG	GCTGCAAAGCGTCTTTCCCTCCG
2	TGGGACGGTGGGGTACAGACTGG	CCGCCTGCTCAGGCTTCCGTGGGG
3	TTCCAGCGAGAGGCAGAGGGA	CGCAGCTCTGCTCGCCCGGCTC
4/Ex1	ACAACACCCGAGCAAGGACGC	GAGCCTTTCAGAGAAGCGGGTCC
5	CCTCGCGCCCGAGATGCGGAG	CAATACGGAGATGCAACTGCGCC
Int1	TATATTCACGCTGACTCCCGGCCG	GCTCAGGATGCAAGGGGCTTT
Ex2	CCAGCTTGTACCTGCAGGATC	CCGAGGACGGAGAGAAGGCGC
Int2	GATTACAGGTGTGAGCCAGG	CACTCCTTTAGCAAGGTTAC
STX4 gene		
1	GGAAGAGACGCGACCATGTGC	CACAGGTTGGAATTCCCTT
2	GCAGAGATCATGGAGTCCAATTGGA	GGGTGTTATAACCGGACTGAGCAT
WBP5 gene		
1	GGCCAAATGTATAAGTGGGG	CCAGGACTTCTGCAC
2	AGGTCTACGGGAACTGATGA	CCGGAAAGCAGTCTTAGATAGC
7SK gene		
	CTGATCTGGCTGGCTAGGCGGG	GAAGACCGGTCCTCCTCTATCGG
CTCF gene		
CTCF	TTCAGGTGGTTAAAGTGGGGGCCAATGGAG	TCCTCTGTATAACGCAGTTTGCTCTTTTTG

### Nuclear run-on

Nuclear run-on analysis was carried out as described ^24^ with 80-nucleotide oligonucleotide probes complementary to RNA transcribed from *U2*. The 3’end of the probes corresponds to positions −130 (probe PSE), +48 (probe R1), +208 (probe R2), +288 (probe R3), +368 (probe R4), +448 (probe R5), +528 (probe R6), +608 (probe R7), relative to the site of transcription initiation. The 3’end of the 80-nucleotide 5S RNA probe corresponding to position +32, relative to the site of transcription initiation was used as a control for the level of transcription. Hybridization signals were quantified by phosphorimager, corrected for the background level (PSE) and normalized to probe 1.

### RNA analysis

RNA was extracted from 6 × 10^6^ control or CTCF K_D_ HeLa cells using TRIzol® (Life Technologies, 15596026, according to the manufacturer's instructions. Reverse-transcription was performed with 1 µg of RNA using oligonucleotides specific and complementary to the sense RNA strand for mRNAs or to 7SK RNA with the SuperScriptIII kit (Life technologies, 11732) according to the manufacturer's instruction. As 7SK RNA levels relative to total RNA were equivalent in control and knockdown samples, these were used for normalization. cDNA was amplified by qRT-PCR using QuantiTect SYBR Green PCR (Qiagen, 204145). Sequences of primer pairs are given in Supplementary data, **Table 1**.

### RNase protection

RNase protection was carried out as described previously.^38^

### Cloning and protein purification

To generate the pFastBac-hisCTCF vector, human CTCF cDNA (Dharmacon, OHS6085-213575129) was amplified by PCR using the following primers (GCGGCCGGATCCGAAGGTGATGCAGTCGAAGCCATTG and GCGGCCGA ATTCTCACCGGTCCATCATGCTGAGGATC), digested by BamHI and EcoRI endonucleases and inserted between BamHI and EcoRI of the pFastBac HT vector (Life Technologies, 10584-02). The pFastBac-hisCTCF vector was used to produce recombinant hisCTCF protein using a Bac-to-Bac® Baculovirus expression system according to the manufacturer's instructions (Life Technologies). His-CTCF purification was performed as follow. Briefly, 3 × 10^7^ infected Sf9 cells were lysed in high salt buffer (50mM Tris pH8, 500 mM NaCl, 1% NP-40, 10 mM Imidazole) by sonication. After centrifugation, the cleared lysate was incubated for 2h at 4°C on a rotating platform with 2 mL Ni-NTA agarose beads (Qiagen, 30210), previously equilibrated in high salt buffer. Beads were then washed twice with TN buffer (20 mM Tris pH 7.9, 500 mM NaCl) containing 10 mM imidazole and 20 mM imidazole and once with NE buffer (20 mM HEPES pH 7.9, 100 mM KCl, 0.2 mM EDTA). Beads were then incubated 4 times with 1 mL NE buffer containing 250 mM imidazole to elute his-CTCF. FLAG-Nelf-E containing NELF complex was purified as described.^28^ FLAG-Spt5 containing DSIF complex was also produced using the Bac-to-Bac® Baculovirus expression system (Life Technologies). GST-NelfA and GST-NelfE were produced as described.^42^

### His-CTCF pull-down

During his-CTCF purification (see cloning and protein purification), 50 uL of Ni-NTA agarose beads bound by his-CTCF were kept after the washing steps and incubated with 250 uL of HeLa nuclear extract for 1h at 4°C on a rotating platform. Beads were washed 8 times with RIPA buffer (50 mM Tris pH8.0, 150 mM NaCl (or 300 mM NaCl as indicated), 1% NP-40, 0.5% sodium deoxycholate, 0.1% SDS, Complete™ protease inhibitor cocktail (Roche, 11697498001)), boiled in Laemmli buffer and analyzed by western blot.

### Recombinant protein pull-down assay

For the FLAG-pull-down assay, 500 ng of his-CTCF and 500 ng of FLAG peptide, FLAG-NELF or FLAG-DSIF were incubated in 200 uL buffer (50 mM Tris pH7.5, 250 mM NaCl, 2 mM EDTA, 0.1% NP-40) for 1 h at 4°C on a shaker. Anti-FLAG® M2 affinity gel (20 uL; Sigma, A2220), previously saturated in bovine serum albumin (BSA), were added to the mixture and incubated for 1h at 4°C on a shaker. Beads were washed 4 times with buffer and samples were eluted 3 times using the FLAG peptide. Eluted samples were boiled in Laemmli buffer and analyzed by western blot. For the GST pull-down assay, 500 ng of GST, GST-Nelf-A or GST-Nelf-E diluted in 100 uL of NP40 buffer (1% NP-40, 50 mM Tris-HCl pH 8.0, 150 mM NaCl) was added to 20 uL of glutathione sepharose 4B (GE Healthcare, 17-0756-01) for 1h at 4°C on a shaker. Beads were washed 4 times with NP40 buffer and resuspended in 50 uL of NE buffer (50 mM Tris pH7.5, 250 mM NaCl, 2 mM EDTA, 0.1% NP-40). His-CTCF (500 ng) was added to the mixture for 1 h at 4°C on a shaker. Beads were washed 5 times with 50 uL of NE buffer, boiled in Laemmli buffer, and analyzed by Western blot.

### Genome-wide analysis

The published datasets for Nelf-E, Spt5, and input reads in HeLa-S3 were obtained from^43^ under the accession number GSE60586. CTCF ChIP-seq and its associated input in HeLa-S3 were obtained from the ENCODE/Broad^44^ under the accession number GSM733785. TSS annotation was acquired from Ensembl GRCh37 release 75.

All sequences were mapped using Bowtie2^45^ version 2.1.0 against the human genome (GRCh37 hg19 from Ensembl). Only uniquely mapped reads were kept and up to 2 mismatches were allowed. Mapped reads were then de-duplicated using Picard to remove PCR-duplicates. Peaks were called with MACS ^46^ version 2.1.0.20150731 using ChIP and input samples and a q-value threshold of 0.01. The CTCF peaks from each replicate were intersected and only the common peaks were kept. The read density around the center of CTCF peaks was generated using HOMER^47^ version 4.7 after normalizing the total number of mapped reads between each sample. *De novo* motif discovery on the 1044 CTCF peaks close to a TSS was performed with the MEME suite MEME-ChIP^48^ using default algorithm parameters.

## Results

### CTCF regulates RNAP II stalling at elongation checkpoints and termination sites

**Figure 1.**
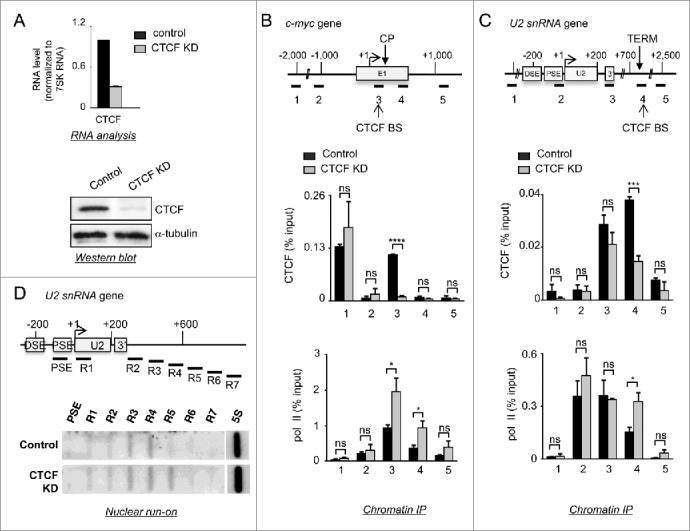
CTCF regulates RNAP II stalling at elongation checkpoints and termination sites. (**A**) RNA (top panel) and Western blot (lower panel) analysis of whole-cell extracts from control cells or cells transfected with siRNA specific for CTCF (CTCF K_D_). The RNA level normalized to RNAP III-transcribed 7SK RNA was quantified by qRT-PCR. Errors bars indicate the standard deviation obtained from at least 3 independent experiments here and in subsequent figures. Antibodies used for Western blot are noted on the right. α-tubulin was used as a loading control. (**B**) ChIP analysis of CTCF and RNAP II occupancy on *c-myc* in control and CTCF K_D_ cells. A diagram of *c-myc* is shown with open boxes to depict exons, an arrow to indicate the transcription start site (TSS), CP to indicate the elongation checkpoint and CTCF BS for the CTCF binding site. The regions amplified by qPCR are noted below the diagram. *P*-values were determined using a non-parametric t-test and indicated as follow: ns for non-significant, * for *P* ≤ 0.05, ** for *P* ≤ 0.01, *** for *P* ≤ 0.001 and **** for *P* ≤ 0.0001 here and in subsequent figures. (**C**) ChIP analysis of CTCF and RNAP II occupancy on *U2* in control and CTCF K_D_ cells. A diagram of *U2* shows the positions of the distal sequence element (DSE) and the proximal sequence element (PSE) in the promoter, the snRNA-encoding region (U2), the 3’ box processing element (3’), the termination site (TERM) and CTCF binding site (CTCF BS). (**D**) Nuclear run-on analysis of *U2* transcription in control and CTCF K_D_ cells. The relative position of single-stranded oligonucleotides used for nuclear run-on analysis is noted under the diagram. An oligonucleotide complementary to transcripts from the RNAP I-transcribed *5S* RNA gene was used as a control for the level of transcription.

Our observation that CTCF binds at sites of Nelf-E association in both protein-coding and snRNA genes ^24^ prompted us to analyze the involvement of CTCF in RNAP II stalling and termination. Accordingly, siRNA-mediated knockdown (K_D_) of CTCF was used to assess its function in expression of the proto-oncogene *c-myc*, as a model of a protein-coding gene where CTCF binds at an early elongation checkpoint and represses expression.^7^ CTCF binds to a site between +5 and +45 downstream from the P2 promoter ^7^ and previous studies have shown that RNAP II pauses between +17 and +52 and that a region upstream of +47 was sufficient to confer promoter proximal pausing.^49,50^ We have also analyzed the effect of CTCF K_D_ on transcription of *U2* as CTCF binds at the point where RNAP II terminates in a NELF-dependent manner, approximately 800 bp downstream from the transcription start site (TSS).^24^ CTCF K_D_ was effective as determined by RNA and Western blot analysis (**Fig. 1A**). Chromatin immunoprecipitation (ChIP) coupled with quantitative real-time PCR (qPCR) determined that CTCF levels are reduced after K_D_ at both the CTCF site just downstream of the *c-myc* TSS (**Fig. 1B**, primer pair 3) and the site at the termination region of *U2* (**Fig. 1C**, primer pair 4). Interestingly, CTCF binding to a site 2 kb upstream of the *c-myc* TSS is not affected (**Fig. 1B**, primer pair 1), suggesting that CTCF has a higher affinity for this site than the one in the transcription unit. CTCF K_D_ does not affect the level of histone H3 (**Figs. S1A and S1B**), suggesting that any effect of CTCF K_D_ on transcription of *c-myc* and *U2* is not due to drastic rearrangements of nucleosomes.

CTCF K_D_ increases the level of RNAP II along the transcription unit of *c-myc* (**Fig. 1B**, primer pairs 3, 4 and 5), while the cellular level of RNAP II is not affected (**Fig. S1C**), suggesting that CTCF is either causing RNAP II to stall at the checkpoint or repressing initiation. The resolution achieved by ChIP (approximately 200 bp) does not allow differentiation between RNAP II at the TSS and the early elongation checkpoint. In agreement with these results, qRT-PCR analysis of mature (mRNA) and nascent (pre-mRNA) transcripts after CTCF K_D_ using primer pairs specific for exonic or intronic sequences shows that levels of both pre-mRNA and mRNA downstream from the checkpoint increase (**Fig. S1D**, primer pairs Ex1, Ex2, Int1 and, Int2). The level of c-Myc protein also appears to increase slightly upon CTCF K_D_ (Figure S1C). These results are in line with a role for CTCF as a repressor of transcription of *c-myc.*^7^

CTCF K_D_ also causes the RNAP II level to increase toward the 3’ end of the *U2* transcription unit (**Fig. 1C**, primer pairs 4 and 5). In accordance with these results, nuclear run-on analysis shows an increase in the levels of nascent transcripts at the end of the *U2* transcription unit after CTCF K_D_ (**Fig. 1D** and **S2**, probes R5, R6, R7). Loss of CTCF causes a defect in termination of transcription very similar to that seen when NELF is knocked down.^24^ The levels of RNAP II and nascent transcripts are instead unchanged on the *STX4* and *WBP5* genes where no CTCF binding sites are located within the transcription unit (**Fig. S3**).^5^ Thus, CTCF helps to stall RNAP II at the elongation checkpoint of *c-myc* and terminate transcription of *U2*.

### CTCF controls NELF and DSIF occupancy on *c-myc* and *U2*

**Figure 2.**
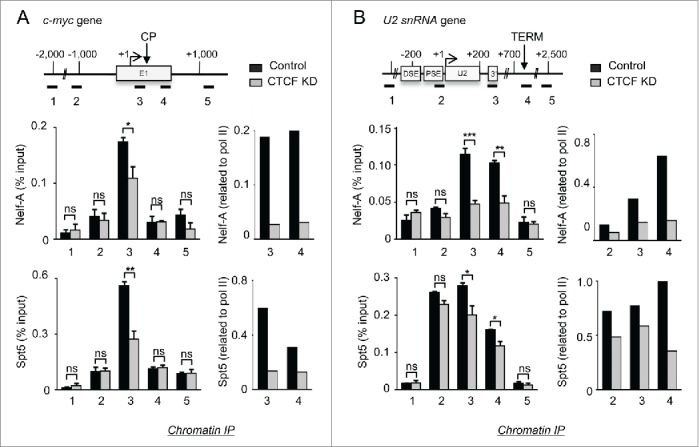
CTCF controls NELF and DSIF occupancy on *c-myc* and *U2.* ChIP analysis of the Nelf-A subunit of NELF, the Spt5-subunit of DSIF and their ratio to RNAP II on *c-myc* (**A**) and *U2* (**B**) in control and CTCF K_D_ cells. The elongation checkpoint (CP) of *c-myc* and the termination site (TERM) of *U2* are indicated.

Since NELF and DSIF are required for RNAP II stalling at the elongation checkpoint of protein-coding genes and NELF controls the termination of transcription of snRNA genes,^24,33^ the effect of CTCF K_D_ on the association of the Nelf-A subunit of NELF and the Spt5 subunit of DSIF with *c-myc* and *U2* was determined by ChIP (**Fig. 2** and **S4**). On *c-myc*, the level of Nelf-A and Spt5 drops after CTCF K_D_ (**Fig. 2A** and **S4A**, primer pair 3), despite an increase in the cellular level of Spt5 (**Fig. S1C**). As NELF and DSIF are recruited to the genes through their interaction with RNAP II, we have also calculated the ratio of Nelf-A and Spt5 to RNAP II. The drop in the ratio of Nelf-A and Spt5 to RNAP II after CTCF K_D_ is even more striking (**Fig. 2A**). Nelf-A association with *U2* is also drastically reduced after knockdown of CTCF (**Fig. 2B**), consistent with the effect of CTCF K_D_ on termination of transcription. The drop in the level of Spt5 upon CTCF K_D_ is however less marked, suggesting an alternative mechanism of recruitment to *U2*. The loss of the negative elongation factors, NELF and DSIF caused by CTCF K_D_ is consistent with a role for CTCF in establishment of the checkpoint on *c-myc* and the loss of NELF caused by CTCF K_D_ is consistent with a defect in termination of transcription of *U2*.

### CTCF controls P-TEFb recruitment/activity and recognition of the U2 3’ box

**Figure 3.**
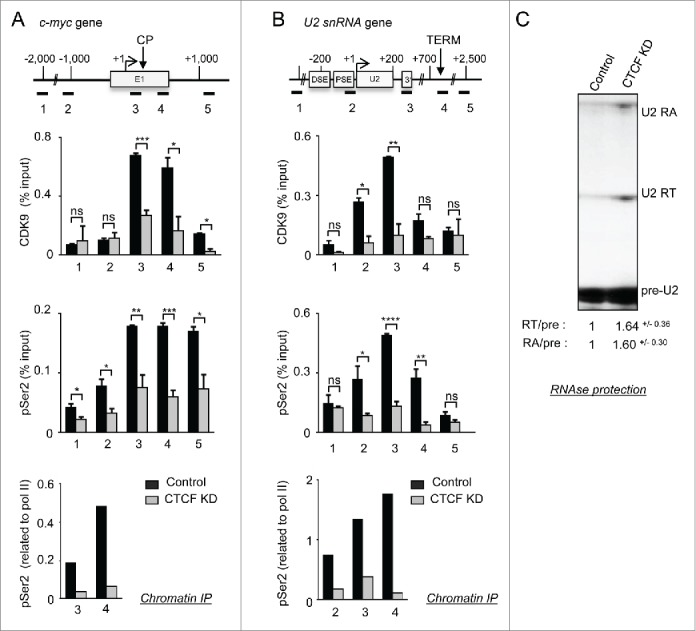
CTCF controls P-TEFb recruitment/activity and recognition of the U2 3’ box. (**A,B**) ChIP analysis of the CDK9-subunit of P-TEFb, phosphorylation of Ser2 (pSer2) of the CTD and pSer2 related to RNAP II on *c-myc* (**A**) and *U2* (**B**) in control and CTCF K_D_ cells. (**C**) RNase protection analysis of transcripts from *U2* in control and CTCF K_D_ cells. The U2 pre-snRNA (pre-U2), the readthrough (U2 RT) and the readaround (U2 RA) are indicated. The ratio of RT/preU2 and RA/preU2 results from the quantification of 3 independent experiments and associated standard deviation is indicated.

We next investigated the effect of CTCF K_D_ on CDK9 and cyclin T1 association with *c-myc* by ChIP (**Fig. 3A** and **S4A**). The level of both proteins peaks in the promoter-proximal region of *c-myc* in control conditions and drop after CTCF K_D_ (**Fig. 3A** and **S4**, primer pairs 3 and 4). As CDK9 phosphorylates Ser2 of RNAP II CTD^34^ and CDK9 recruitment is impaired, we expect a loss of Ser2 phosphorylation upon CTCF K_D_. ChIP analysis indicates that phosphorylation of Ser2 is lower across the promoter-proximal region of *c-myc*, while phosphorylation of Ser5 is much less affected (**Fig. 3A** and **S4A**, primer pairs 3, 4 and 5). CTCF therefore plays a role in recruitment of P-TEFb to this region of *c-myc*. CTD Ser2 phosphorylation may be dispensable for *c-myc* transcription in the absence of CTCF because a checkpoint is not established.

Upon CTCF K_D_, both recruitment of CDK9 and cyclin T1 to *U2* and phosphorylation of CTD Ser2 are impaired (**Fig. 3B**, **S4B**). Phosphorylation of CTD Ser5 is instead largely unaffected (**Fig. S4B**). We have shown that CDK9 is recruited to *U2* and is required for recognition of the snRNA gene-specific 3’ box.^39,51^ As CDK9 recruitment to *U2* is impaired upon CTCF K_D_, 3’ box-directed RNA processing may also be affected. RNase protection of *U2* transcripts detects 3 RNAs, corresponding to pre-U2 snRNA (pre-U2), the read-through product that has escaped 3’ box-directed processing (U2RT), and read around RNA that is produced by RNAP II that has escaped transcription termination and read into the next tandem *U2* (U2RA).^39,52^ The ratio of U2RT to pre-U2 increases when CTCF is knocked down (**Fig. 3C**), indicating that recognition of the 3’ box is affected. U2RA also increases, consistent with a transcription termination defect caused by CTCF K_D_. Thus, CTCF also helps to recruit P-TEFb to *U2* for efficient recognition of the 3’ box recognition.

### CTCF interacts with DSIF, NELF and P-TEFb

**Figure 4.**
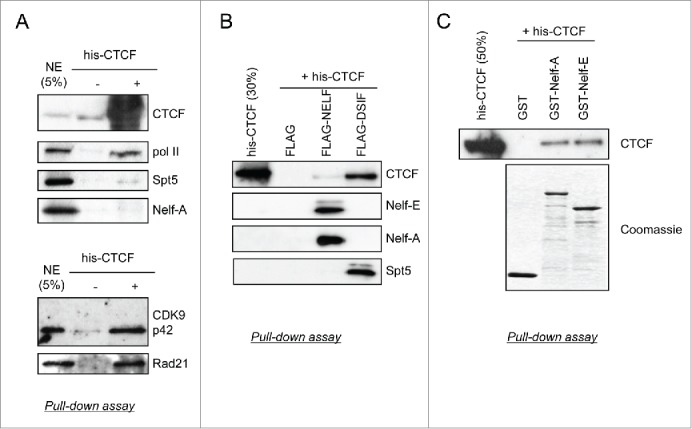
CTCF interacts with NELF, DSIF, and P-TEFb. (**A**) Proteins in HeLa nuclear extract were pulled down using nickel agarose beads previously incubated with his-tagged recombinant CTCF (his-CTCF). The bottom panel shows results obtained with more stringent wash conditions (300 mM NaCl vs. 150mM NaCl for the top panel). Western blot analysis was performed using the indicated antibodies. Nuclear extract (NE) incubated with beads without his-CTCF was used as a negative control. Five percent of NE used for the assay was loaded. (**B**) After incubation with his-CTCF, recombinant FLAG-tagged NELF and DSIF were pulled down using anti-FLAG agarose beads followed by western blot analysis using the indicated antibodies. Incubation of his-CTCF with the FLAG peptide was used as a negative control and 30% of his-CTCF used for the assay was loaded. (**C**) Recombinant his-CTCF was pulled down using glutathione sepharose beads previously incubated with GST (as a negative control) and GST-tagged Nelf-A and Nelf-E. Western blot was performed using CTCF antibodies and recombinant proteins were detected by SDS-PAGE followed by Coomassie staining. Fifty percent of his-CTCF used for the assay was loaded.

As CTCF is required for NELF, DSIF, and P-TEFb recruitment to *c-myc* and *U2*, we have investigated whether CTCF interacts with these factors. Recombinant his-tagged CTCF produced using a baculovirus system was incubated with nuclear extract and the interaction partners were pulled down using nickel beads and analyzed by Western blot (**Fig. 4A**). RNAP II and the rad21 subunit of cohesin, which have been described to interact with CTCF^12,53^ were used as positive controls. The p42 isoform of CDK9 is efficiently pulled-down with his-CTCF and a low level of Spt5 and NelfA are detected. To further investigate the interaction of CTCF with NELF and DSIF, FLAG-Nelf-E-containing NELF and FLAG-Spt5-containing DSIF were incubated with recombinant his-CTCF, pulled-down using an anti-FLAG antibody and analyzed by Western blot (**Fig. 4B**). His-CTCF is effectively pulled down by FLAG-DSIF, and some association with FLAG-NELF is detected. Nelf-A is pulled down with FLAG-Nelf-E, confirming that the NELF complex maintains its integrity (**Fig. 4B**). To further validate the interaction of NELF with CTCF, we performed a GST pull-down analysis after incubation of GST-Nelf-A and GST-Nelf-E with his-CTCF (**Fig. 4C**). His-CTCF interacts with GST-Nelf-A and GST-Nelf-E (**Fig. 4C**). Taken together, our data indicates that CTCF can interact with DSIF and NELF directly and interacts with CDK9 either directly or indirectly. Furthermore, the analysis of available ChIP-seq data for CTCF, Nelf-E and Spt5 ^43,44^ shows an enrichment of Spt5 and Nelf-E binding site at CTCF binding site located at −50bp to +500bp of a TSS (**Fig. S5**), confirming the interaction between these factors.

### CTCF acts upstream of NELF

**Figure 5.**
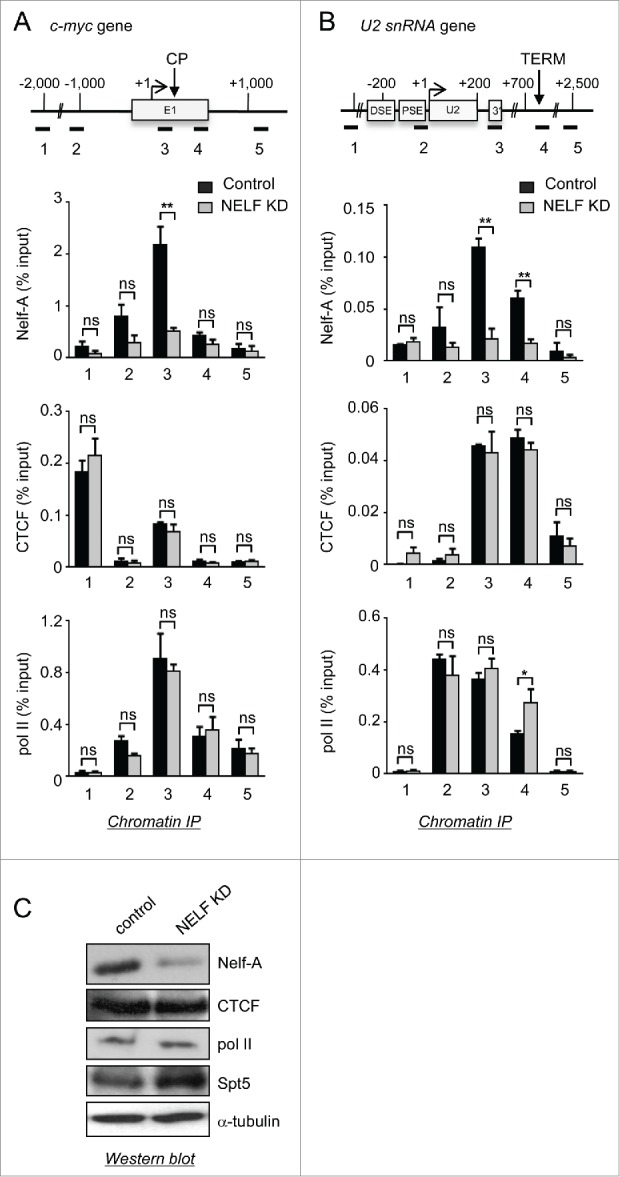
CTCF acts upstream of NELF. (**A,B**) ChIP analysis of Nelf-A, CTCF, and RNAP II occupancy on *c-myc* (**A**) and *U2* (**B**) in control and NELF K_D_ cells. (**C**) Western blot analysis of whole-cell extracts from control and NELF K_D_ cells. Antibodies used are noted on the right. α-tubulin was used as a loading control.

We have shown that CTCF affects the binding/stability of NELF on *c-myc* and *U2*. The next question was whether NELF can in turn regulate CTCF binding. We have investigated the effect of NELF K_D_ on CTCF and RNAP II association with *c-myc* and *U2*. In line with the finding that loss of one subunit of NELF affects stability of the complex,^54^ K_D_ of the Nelf-E subunit efficiently reduces the level of Nelf-A detected by ChIP (**Figs. 5A and 5B**) and Western blot (**Fig. 5C**). ChIP analysis shows that CTCF binding to sites in *c-myc* and *U2* is not affected by NELF K_D_ (**Figs. 5A and 5B**), indicating that NELF is not required to recruit CTCF. In addition, NELF K_D_ does not affect the RNAP II profile on *c-myc* (**Fig. 5A**), suggesting that NELF is not a major negative elongation factor in this context. This is in line with previous findings that, unlike DSIF K_D_, NELF K_D_ does not always have a major effect on RNAP II profiles.^55^

NELF K_D_ does not affect CTCF association with *U2* but a termination defect is observed (**Fig. 5B**), as previously described.^24^ These data suggests that CTCF acts upstream of NELF in the sequence of events that leads to RNAP II termination of *U2*. Western blot analysis indicates that the cellular level of Spt5 increases after CTCF and NELF K_D_ (**Fig. S1C and 5C**), arguing for feedback regulation between these factors.

In conclusion, CTCF helps to recruit and/or stabilize NELF at the end of the *U2* transcription unit to terminate transcription.

### CTCF acts upstream of DSIF and DSIF acts upstream of NELF

**Figure 6.**
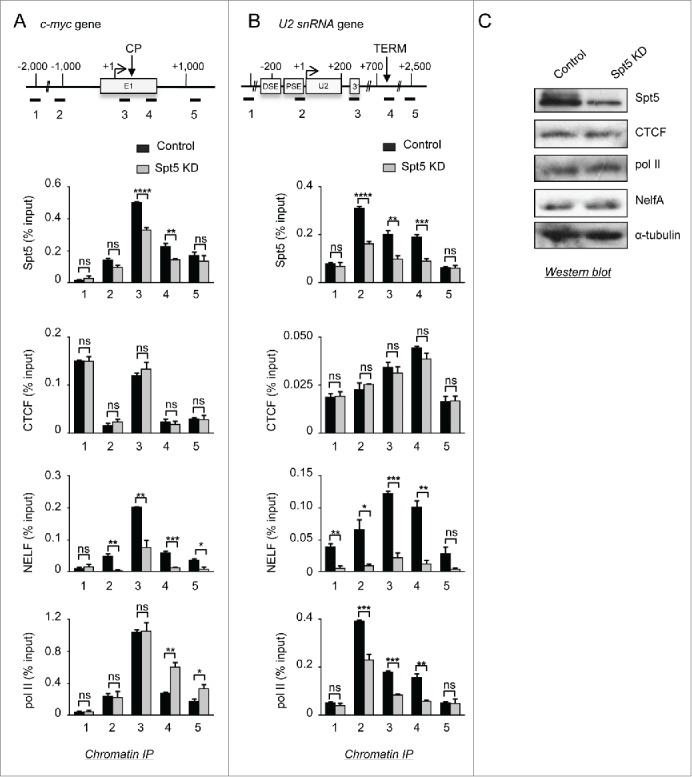
CTCF acts upstream of DSIF and DSIF acts upstream of NELF. (**A,B**) ChIP analysis of Spt5, CTCF, NELF, and RNAP II occupancy on *c-myc* (**A**) and *U2* (**B**) in control and Spt5 K_D_ cells. (**C**) Western blot analysis of whole-cell extracts from control and Spt5 K_D_ cells. Antibodies used are noted on the right. α-tubulin was used as a loading control.

To determine whether DSIF regulates CTCF recruitment, we have assessed the effect of Spt5 K_D_ on CTCF, RNAP II and NELF association with *c-myc* and *U2*. The knockdown efficiency has been tested by ChIP **(Figs. 6A and 6B)** and Western blot (**Fig. 6C**). ChIP analysis shows that CTCF occupancy on *c-myc* and *U2* is not affected by Spt5 K_D_ (**Figs. 5A and 5B**), indicating that DSIF is not required for the recruitment of CTCF. However as expected, the level of NELF on *c-myc* and *U2* is largely reduced upon DSIF K_D_. This is consistent with previous findings that DSIF helps to recruit NELF to the elongation complex.^56,57^

On *c-myc*, the level of RNAP II is increased in the gene body as expected by the loss of the early elongation checkpoint (**Fig. 6A**). DSIF K_D_ has a similar effect to CTCF K_D_ on RNAP II, implicating loss of DSIF in the effect produced by CTCF knockdown. Thus, in the absence of DSIF or CTCF (but not NELF), the elongation checkpoint is not established and RNAP II transcribes *c-myc* without pausing at an early elongation checkpoint.

Conversely, on *U2*, the level of RNAP II decreases along the transcription unit upon DSIF K_D_ (**Fig. 6B**). This result is consistent with the recent demonstration that DSIF is required for efficient transcription of *U1* and *U2.* ^42^ Normalization of the levels of RNAP II at the beginning of the gene indicates that loss of DSIF causes a termination defect, possibly due to the loss of NELF (**Fig. S6**).

**Figure 7.**
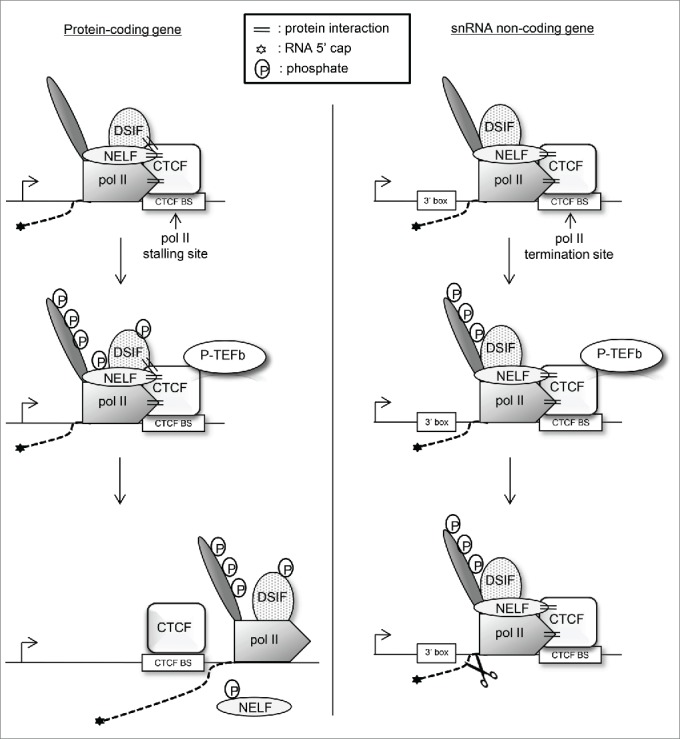
Model for the role of CTCF in the regulation of RNAP II transcription elongation. CTCF bound to its binding site (BS) at early elongation checkpoint or termination sites increases recruitment or stabilization of NELF and DSIF to enhance RNAP II stalling/termination. On protein-coding genes, recruitment of DSIF but not NELF is required for stalling. Recruitment of P-TEFb requires CTCF and phosphorylation converts DSIF from a repressor to an activator, RNAP II phosphorylation activates downstream RNA processing and elongation, whereas phosphorylated NELF leaves the elongation complex. CTCF therefore both help to set up an early elongation checkpoint and allow RNAP II to negotiate the checkpoint while promoting the recruitment of chromatin remodeling, elongation, and RNA processing factors. On an snRNA gene instead, CTCF helps to recruit NELF to cause termination of transcription and P-TEFb to phosphorylate the RNAP II CTD to activate efficient recognition of the 3’ box, thereby linking these 2 processes.

Our current working model, based on the data presented, is that CTCF recruits DSIF and NELF to *c-myc* to first stall transcription while recruitment of P-TEFb promotes the transition to productive elongation. On *U2*, instead, recr-uitment of NELF and P-TEFb by CTCF helps to couple termination of transcription to RNA 3’ end formation (**Fig. 7**).

## Discussion

The location of CTCF-binding sites at sites of RNAP II stalling or termination suggests a role for CTCF in regulation of transcription elongation^7,22-24,49,50^ and 2 recent studies support this. Firstly, CTCF can slow RNAP II down in an *in vitro* transcription system ^21^ and, secondly, genome-wide, CTCF binding to promoter-proximal sites within transcription units is associated with an increase in RNAP II stalling.^20^ However, the molecular mechanism of how CTCF affects transcription elongation and whether CTCF is involved in termination of transcription of snRNA gene was unclear. Our findings indicate that CTCF plays a major role in setting up an early elongation checkpoint on a protein-coding gene and terminating transcription of an snRNA gene through recruitment of NELF, DSIF, and P-TEFb.

CTCF K_D_ causes loss of this factor from its binding sites in the promoter-proximal transcribed region of *c-myc* and the termination region of *U2*. Surprisingly, CTCF binding to a site 2 kb upstream of *c-myc* TSS is not affected, suggesting that CTCF has a higher affinity for the upstream binding site. CTCF helps to recruit and/or stabilize the association of the negative elongation factors NELF and DSIF at RNAP II stalling/termination sites in *c-myc* and *U2.* Accordingly, the reduction of NELF and DSIF levels caused by CTCF K_D_ results in increased RNAP II levels downstream from the CTCF binding sites. CTCF is therefore required for the establishment of the early elongation checkpoint on *c-myc* and termination of transcription of *U2*. Interestingly, on *c-myc*, K_D_ of DSIF, but not NELF, allows productive elongation without affecting CTCF binding. This finding suggests that CTCF helps to establish the early elongation checkpoint on *c-myc* by recruiting DSIF. On *U2*, K_D_ of NELF causes a defect in transcription termination without affecting CTCF binding. This suggests that CTCF plays a role in termination of transcription of *U2* through recruitment of NELF **(Fig. 7)**.

CTCF also helps recruitment of P-TEFb to the elongation checkpoint of *c-myc* and the termination region of *U2.* P-TEFb recruitment will result in the phosphorylation of Spt5, NELF, and the RNAP II CTD, releasing RNAP II from the elongation checkpoint and facilitating efficient transcription elongation and transcription-coupled RNA processing. In line with the impaired recruitment of P-TEFb upon CTCF K_D_, CTD Ser2 phosphorylation is decreased. Thus, CTCF is a core checkpoint factor which controls the amount of RNAP II transcribing through the checkpoint of *c-myc*. CTCF therefore joins the list of factors that recruit P-TEFb to genes, which includes the bromodomain-containing protein 4 (BRD4),^58^ MYC,^55^ nuclear factor-κ B (NFκB) ^59^ and the MED26 component of the Mediator complex.^60^

As CTCF K_D_ causes an increase in RNAP II on *c-myc*, an increase in nascent and steady state transcripts and an increase in levels of the c-myc protein, CTCF is acting as a repressor. In addition, our results suggest that production of mature c-myc mRNA is not strictly dependent on CTD Ser2 phosphorylation at the early elongation checkpoint. GRO-seq analysis indicates that, when CTCF is not knocked down, 2 CDK9 inhibitors, DRB and KM05382, inhibit transcription of c-myc,^26^ suggesting that P-TEFb is required for transcription when CTCF is bound.

Our results indicate that CTCF can interact directly with DSIF and NELF and either directly or indirectly with P-TEFb *in vitro*, highlighting that interactions between CTCF and these factors *in vivo* underlies the effect of CTCF on transcription elongation. The C-terminus of CTCF can be phosphorylated *in vitro* by nuclear extracts and casein kinase II (CKII).^10^ Phosphorylation of CTCF weakens its interaction with RNAP II^12^ and converts CTCF from a repressor to an activator of transcription^13^ in a manner analogous to DSIF phosphorylation.^34^ Phosphorylation of CTCF could also be involved in modulating the interaction between CTCF and other proteins. For example, phosphorylation of CTCF could play a role in releasing RNAP II from the *c-myc* early elongation checkpoint. Furthermore, CTCF-CTCF interactions are known to be involved in the formation of gene loops.^61^ CTCF bound at early elongation checkpoints or termination sites could therefore interact with CTCF bound upstream or downstream to produce chromatin structures refractive to transcription by RNAP II. In this case, CTCF K_D_ would perturb these loops and allow RNAP II to proceed.

Our findings suggest a model where a network of interactions between CTCF, NELF, DSIF, and RNAP II underlies an early elongation checkpoint on some protein-coding genes **(Fig. 7)**. Recruitment of P-TEFb by CTCF will result in phosphorylation of NELF, which then leaves the elongation complex, phosphorylation of DSIF, which is converted into a transcriptional activator and phosphorylation of Ser2 of the RNAP II CTD.^25,32^ In addition, phosphorylation of RNAP II and/or CTCF could affect their interaction.^12^ The network of interactions at RNAP II stalling sites would thereby be weakened and allow the release of the elongation complex from the checkpoint. In addition, the phosphorylation of DSIF and RNAP II (and potentially CTCF itself) also creates a platform to recruit factors involved in chromatin remodeling, elongation, and RNA processing.

CTCF also recruits P-TEFb to *U2*, which activates 3’ box-directed processing of snRNA gene transcripts.^39^ Recruitment of both P-TEFb and NELF by CTCF will help to couple 3’ end formation to termination of transcription (**Fig. 7**). Loss of CTD Ser2 phosphorylation caused by CTCF K_D_ may contribute to the termination defect through the effect on 3’ box recognition.^51,62^ However, it is likely that loss of NELF is the major cause of the termination defect as NELF K_D_ causes a similar defect without affecting RNA 3’ end formation.^24,62^ As CTD Ser5 phosphorylation is also reduced at the end of U2 by CTCF K_D_ this mark could also contribute to efficient termination of transcription.
