# Supplementary material for: CTCF regulates NELF, DSIF and P-TEFb recruitment during transcription
Source: Transcription. 2015 Sep 23;6(5):79–90. doi: 10.1080/21541264.2015.1095269 (PMC4802788; doi:10.1080/21541264.2015.1095269)
Supplement: 1095269_Supplemental_Material.ppt [file ktrn-06-05-1095269-s001.ppt]

## Slide 1
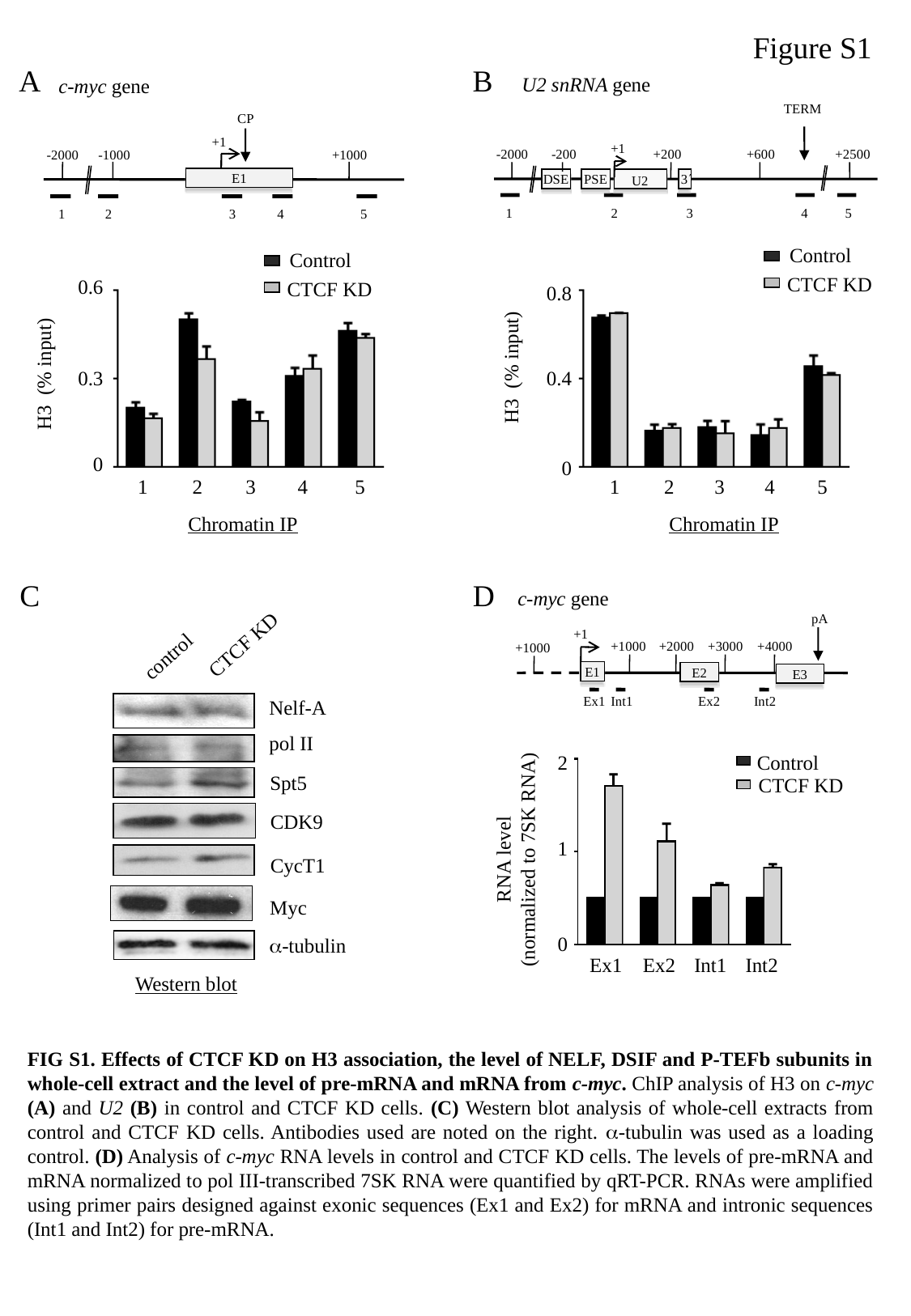

Figure S1
A
B
U2 snRNA gene
c-myc gene
TERM
+1
-2000
-200
+200
+600
+2500
U2
DSE
PSE
 3′
1
2
3
4
5
CP
+1
-2000
-1000
+1000
E1
1
2
3
4
5
Control
CTCF KD
Control
CTCF KD
0.6
0.8
H3 (% input)
H3 (% input)
0.4
0.3
0
0
1
2
3
4
5
1
2
3
4
5
Chromatin IP
Chromatin IP
C
D
c-myc gene
pA
+1
+1000
+2000
+3000
+4000
+1000
E1
E2
E3
Ex1
Int1
Ex2
Int2
CTCF KD
control
Nelf-A
pol II
2
1
0
Ex1
Ex2
Int1
Int2
Control
CTCF KD
Spt5
CDK9
RNA level
(normalized to 7SK RNA)
CycT1
Myc
α-tubulin
Western blot
FIG S1. Effects of CTCF KD on H3 association, the level of NELF, DSIF and P-TEFb subunits in whole-cell extract and the level of pre-mRNA and mRNA from c-myc. ChIP analysis of H3 on c-myc (A) and U2 (B) in control and CTCF KD cells. (C) Western blot analysis of whole-cell extracts from control and CTCF KD cells. Antibodies used are noted on the right. α-tubulin was used as a loading control. (D) Analysis of c-myc RNA levels in control and CTCF KD cells. The levels of pre-mRNA and mRNA normalized to pol III-transcribed 7SK RNA were quantified by qRT-PCR. RNAs were amplified using primer pairs designed against exonic sequences (Ex1 and Ex2) for mRNA and intronic sequences (Int1 and Int2) for pre-mRNA.

## Slide 2
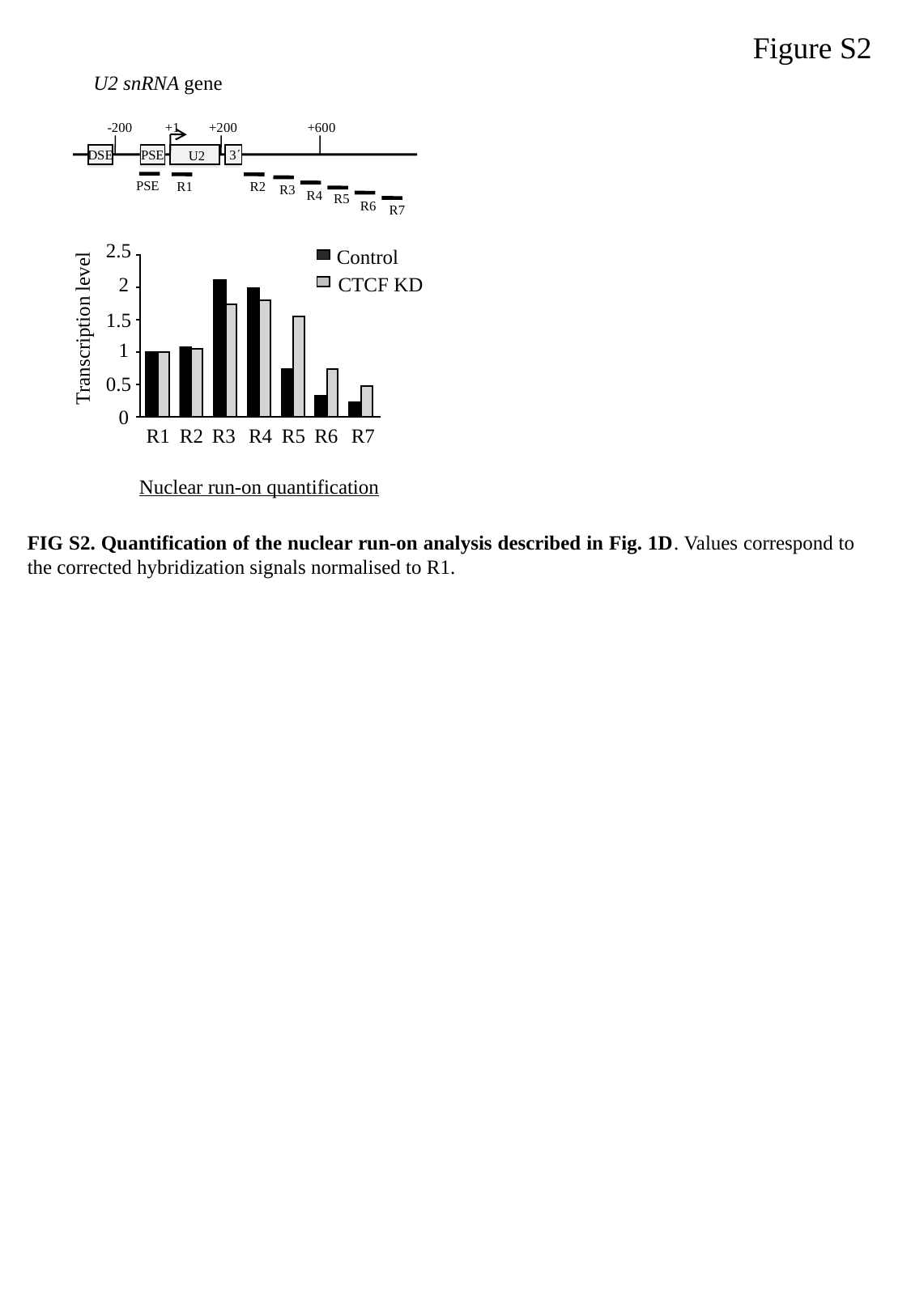

Figure S2
U2 snRNA gene
-200
+1
+200
+600
U2
DSE
PSE
 3′
PSE
R1
R2
R3
R4
R5
R6
R7
2.5
2
1.5
Transcription level
1
0.5
0
R1
R2
R3
R4
R5
R6
R7
Control
CTCF KD
Nuclear run-on quantification
FIG S2. Quantification of the nuclear run-on analysis described in Fig. 1D. Values correspond to the corrected hybridization signals normalised to R1.

## Slide 3
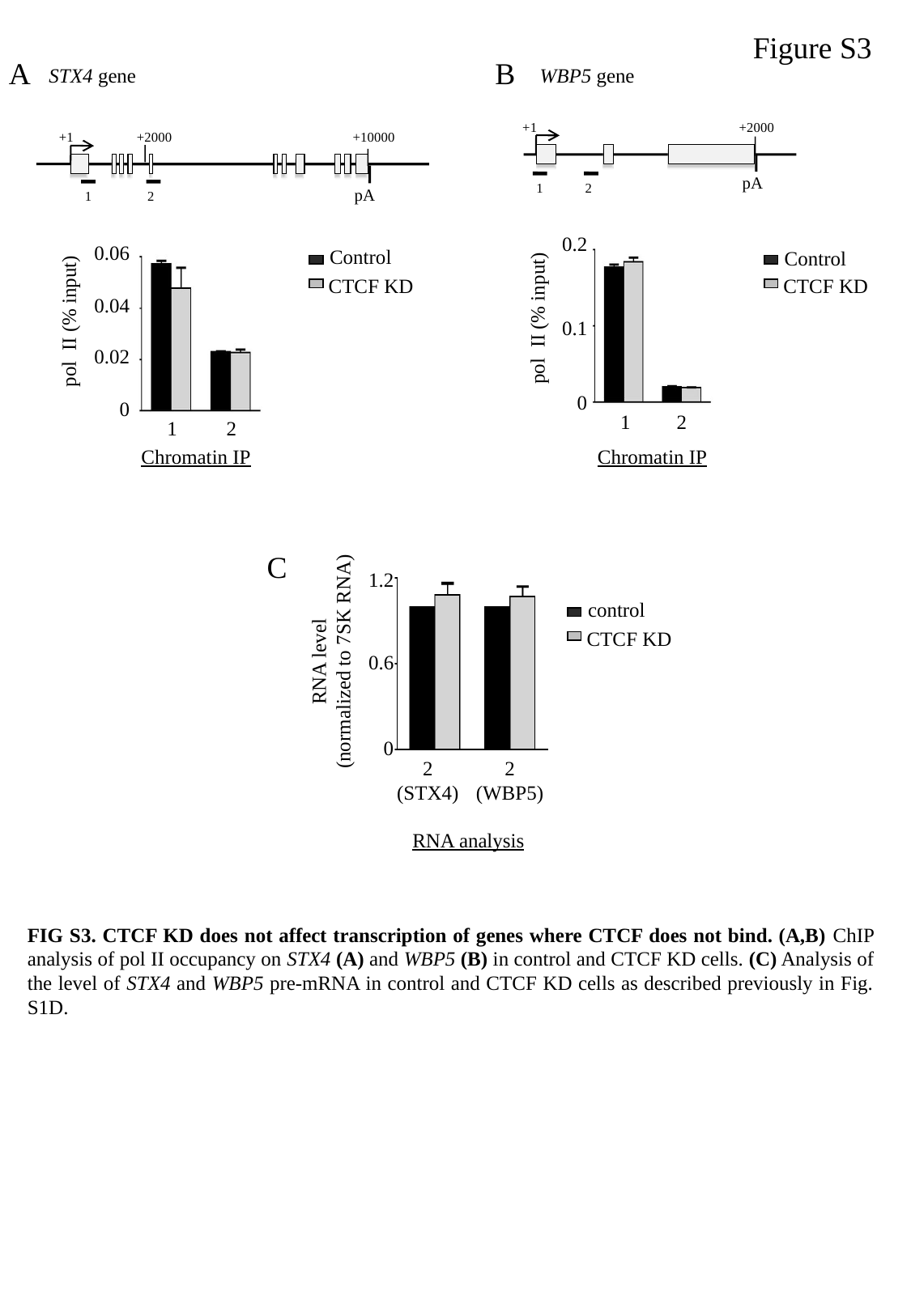

Figure S3
A
B
STX4 gene
WBP5 gene
+2000
+1
pA
1
2
+10000
+1
+2000
pA
1
2
0.2
0.06
0.04
pol II (% input)
0.02
0
1
2
Control
CTCF KD
Control
CTCF KD
pol II (% input)
0.1
0
1
2
Chromatin IP
Chromatin IP
C
1.2
control
CTCF KD
RNA level
(normalized to 7SK RNA)
0.6
0
2
(STX4)
2
(WBP5)
RNA analysis
FIG S3. CTCF KD does not affect transcription of genes where CTCF does not bind. (A,B) ChIP analysis of pol II occupancy on STX4 (A) and WBP5 (B) in control and CTCF KD cells. (C) Analysis of the level of STX4 and WBP5 pre-mRNA in control and CTCF KD cells as described previously in Fig. S1D.

## Slide 4
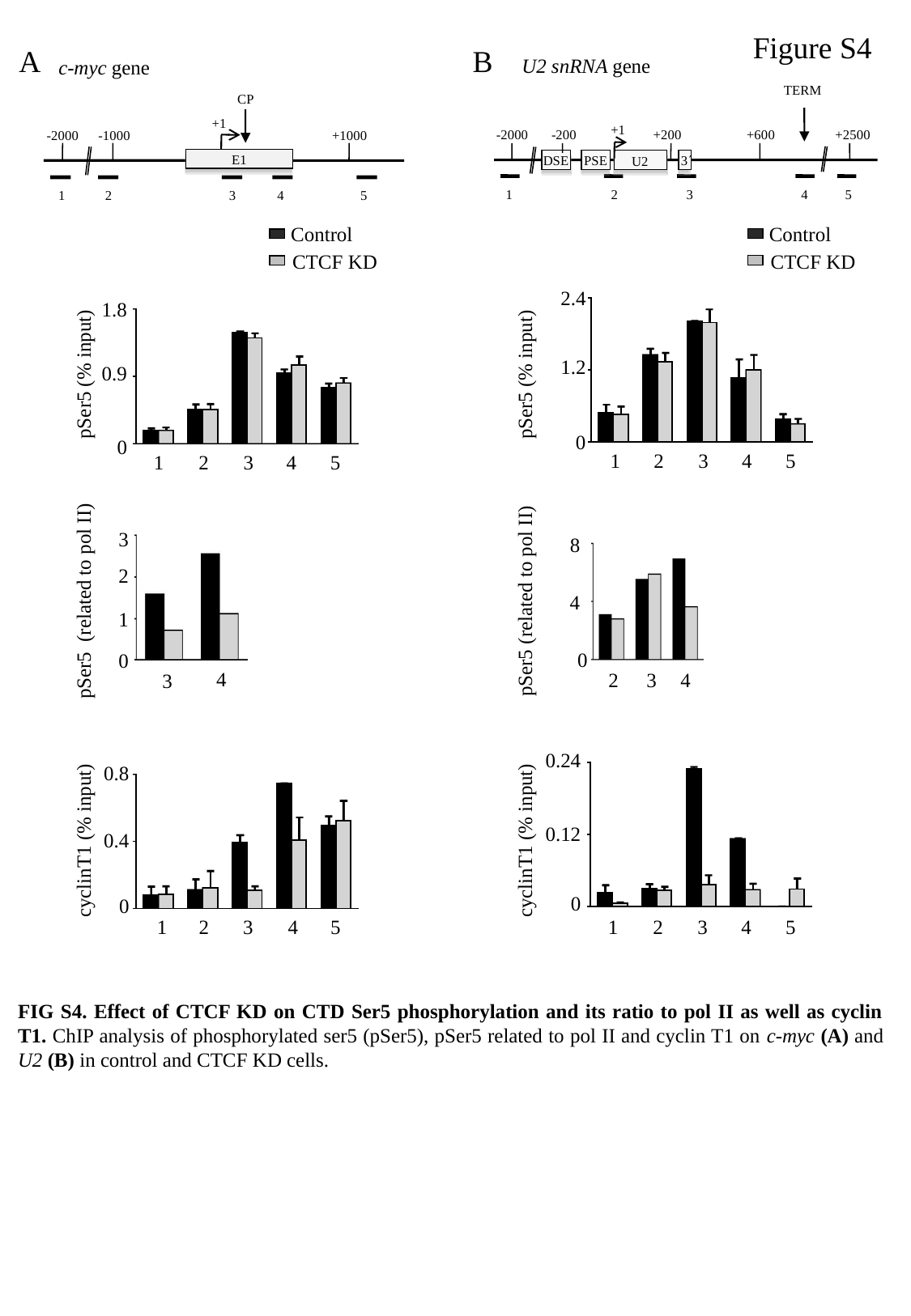

Figure S4
A
B
U2 snRNA gene
c-myc gene
TERM
+1
-2000
-200
+200
+600
+2500
U2
DSE
PSE
 3′
1
2
3
4
5
CP
+1
-2000
-1000
+1000
E1
1
2
3
4
5
Control
CTCF KD
Control
CTCF KD
2.4
1.8
1.2
0.9
pSer5 (% input)
pSer5 (% input)
0
0
1
2
3
4
5
1
2
3
4
5
3
8
2
pSer5 (related to pol II)
pSer5 (related to pol II)
4
1
0
0
4
2
3
4
3
0.24
0.8
0.12
0.4
cyclinT1 (% input)
cyclinT1 (% input)
0
0
1
2
3
4
5
1
2
3
4
5
FIG S4. Effect of CTCF KD on CTD Ser5 phosphorylation and its ratio to pol II as well as cyclin T1. ChIP analysis of phosphorylated ser5 (pSer5), pSer5 related to pol II and cyclin T1 on c-myc (A) and U2 (B) in control and CTCF KD cells.

## Slide 5
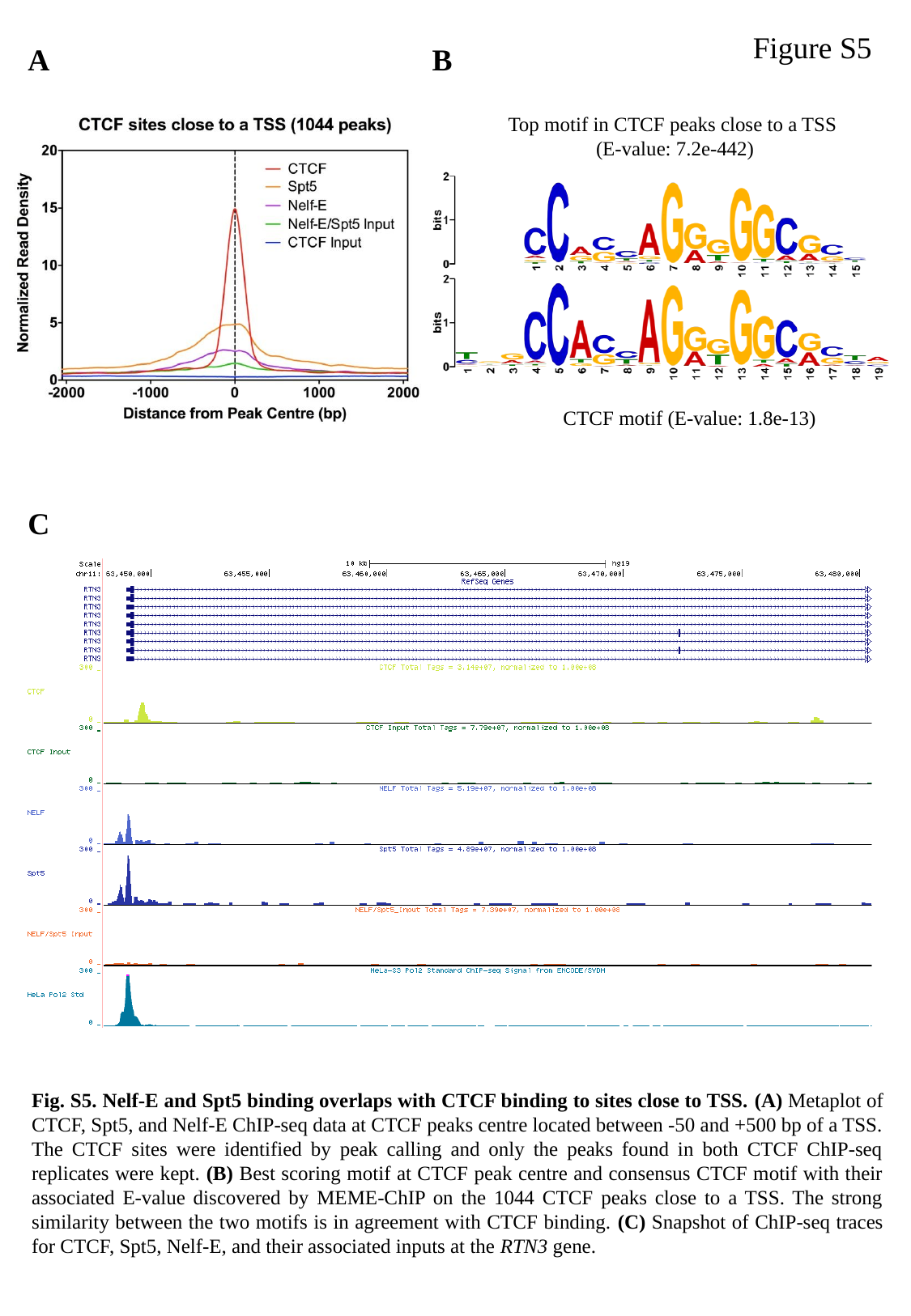

Figure S5
A
B
Top motif in CTCF peaks close to a TSS
 (E-value: 7.2e-442)
CTCF motif (E-value: 1.8e-13)
C
Fig. S5. Nelf-E and Spt5 binding overlaps with CTCF binding to sites close to TSS. (A) Metaplot of CTCF, Spt5, and Nelf-E ChIP-seq data at CTCF peaks centre located between -50 and +500 bp of a TSS. The CTCF sites were identified by peak calling and only the peaks found in both CTCF ChIP-seq replicates were kept. (B) Best scoring motif at CTCF peak centre and consensus CTCF motif with their associated E-value discovered by MEME-ChIP on the 1044 CTCF peaks close to a TSS. The strong similarity between the two motifs is in agreement with CTCF binding. (C) Snapshot of ChIP-seq traces for CTCF, Spt5, Nelf-E, and their associated inputs at the RTN3 gene.

## Slide 6
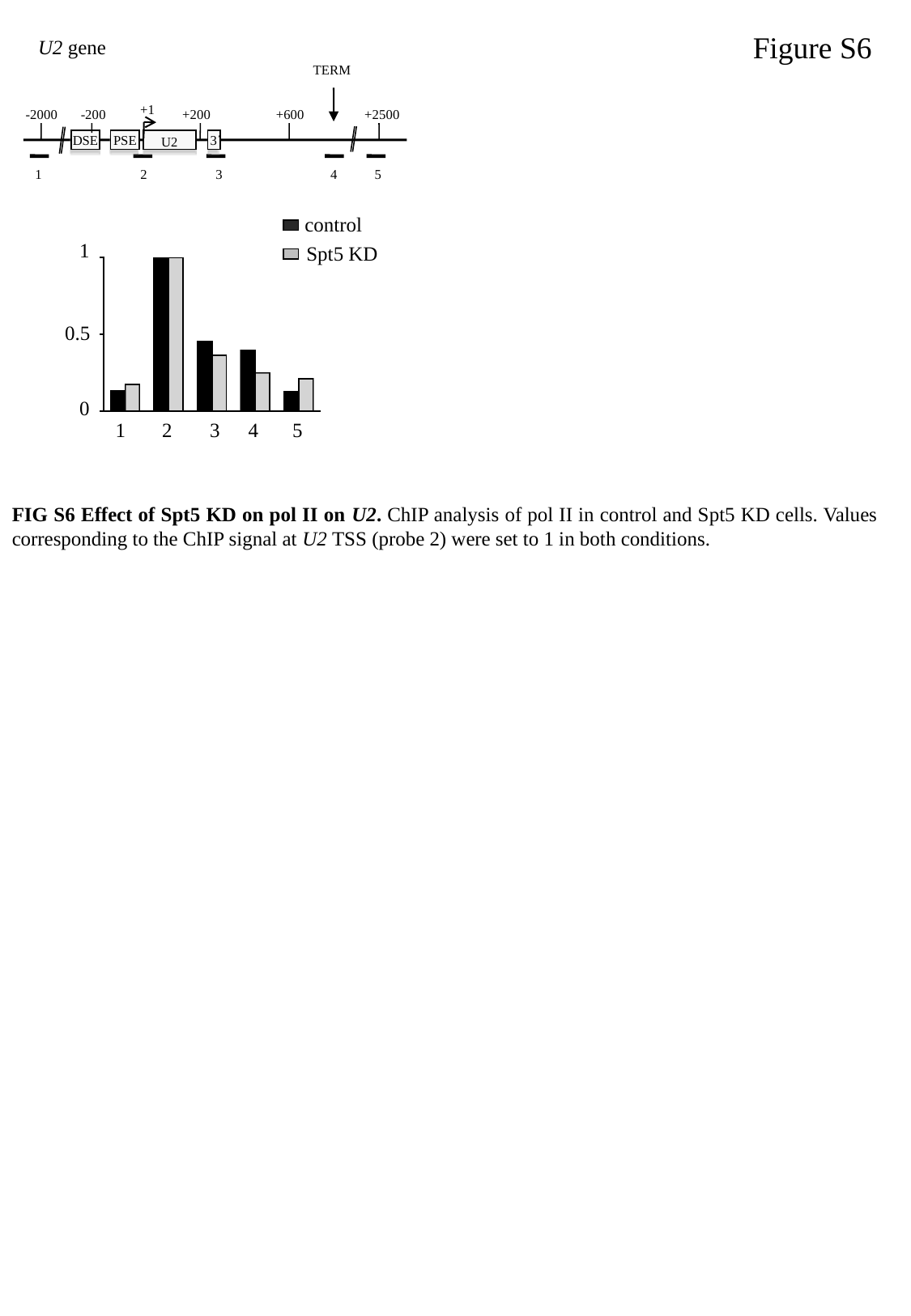

Figure S6
U2 gene
TERM
+1
-2000
-200
+200
+600
+2500
U2
DSE
PSE
 3′
1
2
3
4
5
control
Spt5 KD
1
0.5
0
1
2
3
4
5
FIG S6 Effect of Spt5 KD on pol II on U2. ChIP analysis of pol II in control and Spt5 KD cells. Values corresponding to the ChIP signal at U2 TSS (probe 2) were set to 1 in both conditions.
